# Perceived Barriers to and Facilitators of Physical Activity in Recipients of Solid Organ Transplantation, a Qualitative Study

**DOI:** 10.1371/journal.pone.0162725

**Published:** 2016-09-13

**Authors:** Edwin J. van Adrichem, Saskia C. van de Zande, Rienk Dekker, Erik A. M. Verschuuren, Pieter U. Dijkstra, Cees P. van der Schans

**Affiliations:** 1 Hanze University of Applied Sciences, Research Group Healthy Ageing, Allied Health Care and Nursing, Groningen, the Netherlands; 2 University of Groningen, University Medical Center Groningen, Department of Rehabilitation Medicine, Groningen, the Netherlands; 3 University of Groningen, University Medical Center Groningen, Groningen Transplant Center, Groningen, the Netherlands; 4 University of Groningen, University Medical Center Groningen, Center of Sports Medicine, Groningen, the Netherlands; 5 University of Groningen, University Medical Center Groningen, Department of Pulmonary Diseases and Tuberculosis, Groningen, the Netherlands; 6 University of Groningen, University Medical Center Groningen, Department of Oral and Maxillofacial Surgery, Groningen, the Netherlands; University of Toledo, UNITED STATES

## Abstract

**Background:**

Sufficient physical activity is important for solid organ transplant recipients (heart, lung, liver, kidney). However, recipients do not meet the recommended amount or required type of physical activity. The perceived barriers to and facilitators of physical activity in this population are largely unknown.

**Methods:**

Semi-structured in depth interviews were conducted with solid organ transplant recipients in order to explore experienced barriers and facilitators. Qualitative methodology with thematic line-by-line analysis was used for analysis, and derived themes were classified into personal and environmental factors.

**Results:**

The most important indicated barriers were physical limitations, insufficient energy level, fear, and comorbidities. The most frequently mentioned facilitators included motivation, coping, consequences of (in)activity, routine/habit, goals/goal priority, and responsibility for the transplanted organ. Neutral factors acting as a barrier or facilitator were self-efficacy and expertise of personnel. A comparison of barriers and facilitators between transplant recipient groups yielded no overt differences.

**Conclusion:**

Several personal and environmental factors were indicated that should be considered in intervention development to increase physical activity behavior in solid organ transplant recipients.

## Introduction

In 2014 a total of 7,741 solid organ transplants (SOT) were performed in the EuroTransplant region, resulting in 46.8 transplants per million persons in the entire region and 46.6 per million persons in the Netherlands [1]. Short-term survival after SOT has been greatly improved in the past decades due to advances in organ preservation, surgical techniques, and immunosuppressant medication [2]. As a result, long-term survival and the associated issues like new onset diabetes, medication adherence, and quality of life have gained increased attention, as has the level of physical activity (PA) after SOT.

While clinical experience and a few small studies indicate that SOT recipients can achieve average to above average levels of PA [3–5], the majority of recipients do not meet the recommended amount and type of PA [6–14]. Movement behavior is below the levels of the average population, which results in a generally sedentary and inactive lifestyle. This occurs despite the fact that a higher level of PA has been shown to be associated with decreased cardiovascular and all-cause mortality in renal transplant recipients and improved outcomes like a shorter hospital stay and increased short-term survival in lung and liver transplant recipients [2]. Furthermore, exercise training has been shown to improve physical functioning and quality of life in SOT recipients [15,16] and has the potential to reduce cardiovascular risk factors [15].

It is not fully clear why solid organ transplant recipients do not regain a normal level of physical activity, but several physical factors are likely to contribute to the low PA levels in this population. Firstly, peripheral muscle dysfunction exists pre-transplantation in all organ recipient groups [2,17]. This condition is aggravated post-transplantation by many factors including the use of immunosuppressive medication [17–19]. Secondly, a reduction in VO_2_ peak ranging from 20–50% is observed despite near normal functioning of the transplanted organ [17].

In general, several personal and environmental factors may also influence PA behavior. However, perceived barriers to and facilitators of PA in SOT recipients are primarily unexplored. Barriers refer to perceived obstacles that hinder the performance of PA, and facilitators refer to factors increasing the likelihood of performing PA [20]. Several barriers to and facilitators of PA are indicated in end-stage kidney, liver, lung, and heart disease. Salient barriers in the end-stage disease populations being indicated are ‘fluctuating health status’, ‘concerns about aggravation’, ‘fatigue’, ‘shortness of breath’, ‘fear of falling’, ‘lack of support’, and ‘lack of motivation’ [21–28]. Important facilitators being indicated in the end-stage disease phase are ‘exercising for health’, ‘social support’, ‘professional support’, ‘enjoying the activity’, ‘control of the condition’, and ‘social interactions’. While several barriers to and facilitators of PA in the transplant population have been proposed, only one study has examined them in a single group of SOT recipients by means of a questionnaire [29]. Examples of important barriers ascertained in kidney transplant recipients are ‘lack of motivation’ and ‘preferring to spend time otherwise’, whereas important facilitators were ‘feeling healthy’ and ‘wanting to feel better’. However, the use of the questionnaire did not provide insight into the recipients’ experiences of these barriers and facilitators and was limited to only kidney transplant recipients. The most appropriate research technique indicated in order to get insight into the individual’s decision making process and to get detailed information on perceptions, beliefs and attitudes is the use of individual semi-structured interviews [30]. The aim of the current qualitative study was to explore the perceived barriers to and facilitators of PA in the four major SOT recipient groups.

## Materials and Methods

### Participant selection

Semi-structured interviews were performed with patients who had received a solid organ transplantation at the University Medical Center Groningen (UMCG). Participants were eligible if they had a stable organ function, and if they comprehended the Dutch language. A purposive sampling strategy was employed in which participants were selected who would adequately represent diversity and produce relevant information to the research question (i.e., active vs. inactive). Judgment of suitability was made by the treating physician or physician’s assistant on the basis of their impression and anamneses of their latest contact with the patient in the outpatient setting; the exact level of physical activity was not assessed. Suitable participants were approached by their treating physician or physician’s assistant and were invited to return an enrollment sheet by mail after reading the invitation letter. Interviews occurred in blocks of four participants in order to maintain an equal distribution of four organ groups (heart, lung, liver, and kidney). Interviews were conducted until data saturation occurred (additional interviews to the point at which no new themes arose during subsequent data collection). All participants provided their written informed consent prior to the interview. Data were coded and anonymized. The study complied with the declarations of Helsinki and Istanbul. None of the transplant donors were from a vulnerable population and all donors or next of kin provided written informed consent that was freely given. The Institutional Review Board of the UMCG provided approval to conduct the study (METc 2013/410).

### Data collection

Individual in-depth interviews were performed by one investigator (EA), a human movement scientist/ physical therapist (aged 30, male) with four and a half years of experience with transplant recipients in the inpatient and outpatient setting. Interviews took place between December 2013 and January 2014 and were carried out at the participant’s home or during an outpatient visit at the convenience of the participant. The interviewer had no treatment relationship with any of the interviewed participants. The areas addressed in the interviews were outlined in an interview guide with open-ended questions covering themes and items derived from the literature, a pilot study, and key questions from physical therapists, clinicians, and researchers (S1 Appendix). Thereby, PA was defined as any bodily movement produced by skeletal muscles that resulted in energy expenditure [31] and as a guideline for participants this was formulated as activities that made the breathing frequency go up and resulted in getting warmer or sweating. The interview guide was tested and evaluated in a pilot setting with two organ transplant recipients not participating in the actual study, after which final alterations were made. The content of the interview guide could be revised during the process, and additional questions could be added in order to address newly emerged topics derived from the ongoing data analysis. The interviews were recorded and transcribed verbatim.

### Data analyses

**Fig 1:**
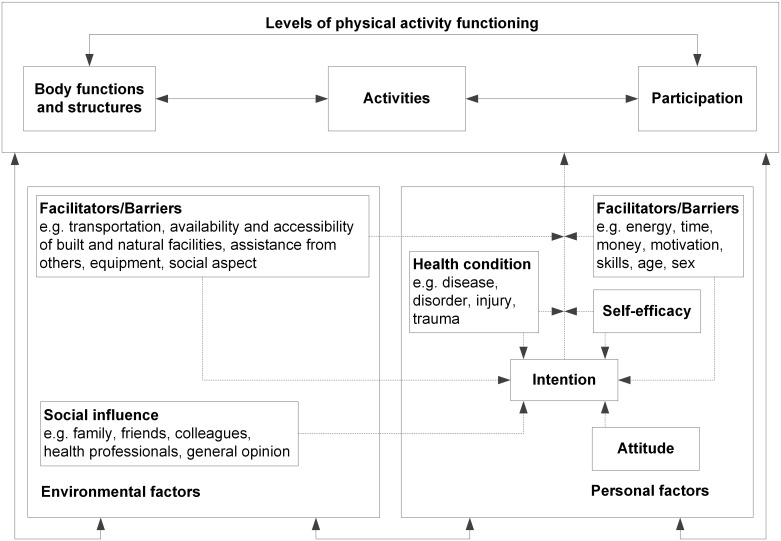
Physical Activity for People with a Disability (PAD) model. Reprinted from van der Ploeg et al., 2004 [34] under a CC BY license, with permission from Springer, original copyright 2004.

The data were analyzed using line-by-line thematic analysis [32,33]. First, two individual reviewers (EA and SZ) familiarized themselves with the raw data after which initial codes were generated of interesting features of the data. The data from the first four interviews were reviewed and coded independently by the two reviewers, and inconsistencies were reviewed and discussed with a third reviewer (PD). A codebook was subsequently created which the two reviewers used to independently code the remaining interviews. The codebook was used as a guide, however, new codes could be given as well. After completing the coding of interviews, the reviewers compared results, and consensus was reached. The codes used were subsequently collated into potential themes, which were reviewed by checking them against the coded extracts and the original data. Initially, all themes were placed in a scheme to add insight into themes being absolute barriers, absolute facilitators, or being positioned in between this continuum, and to clarify which themes were stronger or weaker. A five category scale was chosen indicating (1) absolute barriers; (2) themes being mentioned mainly as barriers but sometimes as a facilitator; (3) themes being mentioned as a barrier or facilitator equally; (4) themes being mentioned mainly as facilitator but sometimes as a barrier; and (5) absolute facilitators. The themes were subsequently ordered into *personal factors* and *environmental factors* as part of the Physical Activity for people with a Disability (PAD) Model (Fig 1) [34]. This model is based on the International Classification of Functioning (ICF), Disability and Health and the Attitude, Social Influence and Self-Efficacy (ASE) Model and was selected because of its integration of the concept of PA behavior and its relationship with functioning. Although the PAD Model was developed as a theoretical framework on PA of people with a disability, both underlying models (ICF and ASE model) were developed for the general population and are not disease specific. The content of the PAD model was judged as generic enough to be suitable for the population under study. After classification in the model, definitions and names for each theme were clarified, and quotes (given in italic font) were selected that illustrated the derived themes [32]. Peer debriefing (review by field expert) secured quality assessment. In the process, the qualitative data analysis program ‘ATLAS.ti’ was used to manage, organize, store, and annotate the data (ATLAS.ti, v 6.2.23, Scientific Software Development GmbH, Berlin, Germany).

## Results

During the study period, 16 potential participants were approached, and all were willing to participate. There were four recipients from each organ transplantation group. Participant characteristics are presented in Table 1.

**Table 1:** Participant characteristics.

	Transplanted organ	Disease before transplantation	Time since Tx (months)	Gender (M/F)	Age (years)	Immunosuppressive medication	Comorbidities
1	Heart	DCM	93	M	51	Tacrolimus, mycophenolate mofetil	DM II
2	Heart	DCM	63	M	41	Prednison, ciclosporine	-
3	Heart	HCM	43	F	56	Prednison,tacrolimus, mycophenolate mofetil	Hyperthyroidism
4	Heart	Cong. AV-block	23	F	21	Tacrolimus, mycophenolate mofetil	-
5	Lung	COPD	6	F	57	Prednison,tacrolimus, mycophenolate mofetil	HT
6	Lung	PH	8	F	50	Prednison, tacrolimus	Reduced renal function
7	Lung	PH	12	M	53	Prednison,tacrolimus, mycophenolate mofetil	Hepatitis C, hypercholesterolemia, reduced renal function
8	Lung	COPD	11	M	39	Prednison,tacrolimus, mycophenolate mofetil	Hypercholesterolemia, reduced renal function
9	Liver	Autoimmune hepatitis	18	M	70	Prednison,tacrolimus, mycophenolate mofetil	DM II
10	Liver	PSC	19	M	43	Tacrolimus, mycophenolate mofetil	-
11	Liver	PBC	144	F	63	Prednison	Reduced renal function
12	Liver	Autoimmune hepatitis	123	M	22	Prednison,tacrolimus, mycophenolate mofetil	Osteoporosis
13	Kidney	IgA nephropathy	15	M	45	Prednison, mycophenolate mofetil	HT, hypercholesterolemia
14	Kidney	Amyloidosis	6	M	54	Prednison,tacrolimus, mycophenolate mofetil	Amyloidosis, hyperthyroidism
15	Kidney	Hypertensive emergency	7	F	42	Prednison,tacrolimus, mycophenolate mofetil	-
16	Kidney	Renal cysts	11	F	52	Prednison, mycophenolate mofetil, ciclosporine	Sjögren
	Median		16.5	9M/ 7F	50.5		
	IQR		8.8; 58.0		41.3; 55.5		

PBC, primary biliary cholangitis; DCM, dilating cardiomyopathy; COPD, chronic obstructive pulmonary disease; PH, pulmonary hypertension; PSC, primary sclerosing cholangitis; HCM, hypertrophic cardiomyopathy; DM II, diabetes mellitus type II; HT, hypertension; IQR, interquartile range.

Several barriers and facilitators to being physically active were identified from the interviews and coded accordingly (Table 2). No overt differences were determined in the distribution of the mentioned barriers and facilitators between the different SOT recipient groups (S1 Table).

**Table 2:** Overview distribution barriers to and facilitators of physical activity and solidity in the data.

	Barriers	←		→	Facilitators
	1.	2.	3.	4.	5.
Stronger	Physical limitations (15/75)	Energy level (12/45)		Coping (15/55)	Motivation (16/148)
↑	Fear (9/20)		Self-efficacy (13/35)	Routine/habit (14/40)	Consequences of (in)activity (14/40)
	Comorbidity (8/26)		Expertise of personnel (13/26)	Goals/goal priority (13/88)	
	Bad weather (6/8)			Transplanted organ (10/29)	
↓	Post-transplant life-events (4/6)	Side-effects medication (6/12)		Social support (9/14)	
	Age (3/4)	Social role (5/19)		Strength (8/15)	
Weaker	Financial resources (3/4)		Group activity (4/8)	Weight (5/13)	
					

Codes are classified on the continuum from barrier to facilitator (1. [red] absolute barriers; 2. [orange] themes being mentioned mainly as barriers but sometimes as a facilitator; 3. [yellow] themes being mentioned as a barrier or facilitator equally; 4. [light green] themes being mentioned mainly as facilitator but sometimes as a barrier; and 5 [green] absolute facilitators) and on the continuum of being a stronger or a weaker factor. The numbers in parenthesis indicate the number of interviews the code is represented in and the groundedness of the code (number of quotations linked to that particular code).

All of the identified barrier and facilitators were classified in the PAD Model. Personal factors were divided into *physical*, *psychological*, and *other* factors, and environmental factors were divided into *social environment*, *physical environment*, and *other* (Table 3).

**Table 3:** Overview of personal and environmental barriers and facilitators to physical activity in recipients of solid organ transplantation.

**Personal**	*Barriers*	*Facilitators*
*Physical*	Physical limitations	
	Lack of energy	Increased energy level
	Comorbidity	
	Lack of strength	Maintaining/increasing muscle strength
	Weight being a barrier	Wanting to lose weight/maintain weight
	Age	
*Psychological*	Lack of motivation	Motivation
	Lack of time	Reinforcement
	Other priorities	Fun/pleasure
	Fear (complications/injury)	Competition
	Coping	Coping
	Goals/goal priority	Goals/goal priority
	Post-transplant life-events	Self-efficacy
*Other*	Routine/habit	Routine/habit
		Consequences of (in)activity
		Transplanted organ
**Environmental**	*Barriers*	*Facilitators*
*Social environment*	Lack of expertise personnel	Expertise personnel
	Lack of social support	Social support
	Lack of group activity	Family/friends
	Social role	Professionals
		Group activity
*Physical environment*	Bad weather	Having a dog (motivation)
*Other*	Side-effect medication	-
	Financial resources	

### Personal barriers and facilitators to PA

#### Physical

The most salient physical barrier mentioned was having physical limitations. Physical limitations refer to inadequate physical capacity to perform PA such as insufficient exercise capacity or muscle strength and body signals like pain or cramps. These physical limitations were most often described in relation to the transplantation but were indicated as unrelated to the transplantation by some.

‘So, it’s mainly due to the loss of coordination, that’s the hardest part. When kicking a ball and standing on one leg, stuff like that, then you experience a barrier.’

(LungTx, male, 53 years)

The aspect strength was indicated in half of the interviews and was described in relation to experienced physical limitations. Inadequate strength to perform activities was experienced as a motivator to perform strength training by some but was experienced as a barrier to be physically active by others.

‘I really had a lot of need for strength training. I noticed that my entire body had actually failed. I needed to do some muscle building’

(HeartTx, male, 51)

Comorbidities were also mentioned in relation to experienced physical limitations. As comorbidities limited the physical capacity or interfered with PA behavior in another way they were only mentioned as barriers.

‘So, I had some problems with my heart, chronic atrial fibrillation. And once in a while it really seriously skipped a beat and then it frightened me a little. So I was afraid of it.’

(LungTx, male, 53 years)

The majority of participants mentioned their energy level. Energy level was primarily stated as a barrier to PA but was described as a facilitator as well. A lack of energy to perform PA was usually mentioned in combination with goal priority. Other social activities or obligations were preferred instead of PA. Some participants perceived that being physically active could also lead to a feeling of increased energy and thereby be facilitating.

‘I’m kind of in a dilemma, because when I use my energy for school I cannot use it for sports.’

(LiverTx, male, 22 years)

Finally, participants mentioned weight as a personal physical barrier or facilitator. Being overweight was a barrier to some due to a perceived increased threshold for initiating being active. Others indicated that maintaining or reducing weight was a facilitator for being active, which was often related to the suspected relationship between medication use and weight gain.

‘Look, I have gained weight enormously and it is not like it is limiting me in my daily life, not really, but I do have a higher threshold towards sports engagement.’

(HeartTx, male, 51 years)

#### Psychological

The most mentioned psychological barrier and facilitator to PA was motivation. Aspects of motivation that were mentioned could be divided into *reinforcement* (the perception of health benefits related to physical activity), *fun/pleasure*, and *competition*. Perceiving health benefits was mentioned as a facilitator and reinforced participants to maintain or increase PA levels. Perceiving fewer health benefits was indicated as resulting in a lack of reinforcement.

‘The satisfaction… you are building something, that’s it. The first weeks you are exhausted and not able to do that much. But then you are just…, you do a warming-up and then you do some strength training and cool down on the rowing machine. And that with another heart, you know, that is so strange.’

(HeartTx, male, 41 years)

Having fun and experiencing pleasure while being physically active and finding pleasure in competition are mentioned as facilitators of PA.

‘Well, I did sports before; it really is one of my passions. I just think it’s very important. I wasn’t able to do it for four years and now that I can I want to make full use of it.’

(HeartTx, female, 21 years)

A lack of motivation is mentioned as a barrier to PA. Aspects related to a lack of motivation that were indicated were a lack of time and having other priorities. These later aspects were mentioned in relationship to goal priority and energy level as well.

‘Yes, I used to do it [training] but on a given time it became gradually less frequent until I stopped. I think I should train but I have to overcome so much resistance, so, is that worth it?’

(LiverTx, female, 63 years)

Goals or goal priority were indicated as a barrier as well as a facilitator. Participants described that when prioritizing other aspects of life over PA, they function as a barrier. As previously stated, this was also mentioned in combination with insufficient energy and/or a lack of motivation. It was indicated that having goals for which a certain exercise capacity is required can facilitate PA behavior.

‘‘I have to get to the point on which I say, “now I have to change course”. But now is not the time…’

(HeartTx, male, 51 years)

‘The moment you start noticing you can handle physical things again, then you start planning. And then you start the list…, well, it’s not like I had a bucket list of things I wanted to do before I died. But, for me, at that time, the list started of all the things I would have done when I wouldn’t have been ill. And I needed to train for these goals.’

(HeartTx, female, 56 years)

Other psychological barriers to or facilitators of PA that were mentioned are coping with post-transplantation life events and self-efficacy. Participants’ coping style was indicated as being facilitating or function as a barrier. The latter is the case with post-transplant life events. A high level of self-efficacy can provide participants with a certain control over life through which they are able to cope with situations.

‘I honestly have to say… it suits me, as a person. To do sports is an outlet. I clear my mind. Somebody else might cry about it but I go out and do sports instead.’

(LiverTx, male, 43 years)

‘[After I was transplanted] I lost a good friend who had the same disease as I had, she died on my birthday…That gave me the biggest setback, then it started to take its course. I actually couldn’t recover from that.’

(HeartTx, male, 51 years)

The final psychological aspect mentioned as a barrier is *fear*. This aspect is presented in combination with the aspect *transplanted organ* in the subsequent section.

#### Other

Personal aspects categorized in the division *other* were routine/habit, consequences of (in)activity and the transplanted organ. PA being a routine or a habit of a participant was mentioned as a facilitator to participation in PA. A routine without PA was indicated as being so habitual in a participant that changing it is very difficult.

‘Well, I don’t really like fitness, I would rather go biking outside or go for a walk. But I do it anyway because, on the one hand I can be compulsively, and it keeps me on track.

(HeartTx, female, 56 years)

‘I haven’t done anything for fifteen, twenty years. So yes, that… So, everything has to…, for everything you have to…not feel like it, but do it. But, because you didn’t do it for such a long time…’

(LungTx, male, 53 years)

Knowledge regarding the physical or medical consequences of being inactive and knowing the benefits of being active were both perceived by participants as facilitators of PA behavior *(consequences of (in)activity)*.

‘I’m the type of person, when somebody says it is good for you to be physically active because you would possibly have an increased risk of heart disease, diabetes or other things due to the medication that you use then I have that in the back of my mind. Then I think, yes, I can sit here comfortably but I better get moving.’

(KidneyTx, male, 54 years)

An indicated facilitating aspect specific for the population under study was the transplanted organ. Having received a transplantation and wanting to take good care of the new organ was described as a facilitator to PA. The sheer fact of having the ability to be physically active was mentioned as facilitator as well.

‘Especially with biking and so on. Because you have another kidney, I want to live longer, healthier, all off that plays along.’

(KidneyTx, female, 42 years)

‘For me it is very, but really very connected to each other. You know, like my life after the transplantation is a life in which I can be active, so I am.’

(HeartTx, female, 56 years)

Receiving a transplantation is not mentioned as a barrier, per se, but a number of factors classified under the psychological personal barrier *fear* are related to the transplanted organ. Fear of damaging the new organ and insecurity with the body and body signals were indicated as a barrier.

‘Yes, look, when I would go cycling into the forest, what I would like to do, but then you are alone… And you think, what if something happens, well… it might take a day before they find me. So I do not,… no I do not dare to.’

(KidneyTx, male, 45 years)

### Environmental barriers and facilitators to PA

#### Social environment

The most salient aspect of the social environment mentioned as a barrier as well as a facilitator is the expertise of personnel. Inadequate expertise was identified as a factor that could incite a negative feeling about being physically active and even influence people to discontinue training. Sufficient expertise, on the other hand, was indicated as a great stimulant. Learning how to train and becoming educated regarding their new boundaries in a controlled setting was described as a stimulant to be physically active.

‘There was a program set up [by the local physiotherapist] and the only thing I remember is that it made me completely worn out. One day, another therapist took over and asked what part of the program I did today and what part at the other session of the week. I said I had to do the entire program, but he said that it was way too much. So it was split in two and since then I started to make progress.’

(LiverTx, female, 63 years)

‘The physical therapist provided excellent help. It was especially helpful in improving your self-confidence. And that was very useful afterwards with fitness training and all.’

(HeartTx, female, 56 years)

Furthermore, participant perceived that PA behavior was influenced through social support and by exercising in a group. Family and friends were indicated to motivate or function as an exercise partner. Professionals also were mentioned to have a stimulating role by providing a pleasant environment in which to exercise. Exercising in a group was perceived as motivating because of the social contact as well as a certain experienced obligation to be present. These factors were all indicated also as barriers.

‘I could ride [my bicycle], sitting behind the other boys [down the wind] and they kept an eye on me. So that is a good thing, when you have friends that guide you a little.’

(LiverTx, male, 43 years)

‘Because you are there [to exercise] for quite some time already you know everybody [all therapist], that makes it more pleasant. The social aspect, that is what I like about it too.

(KidneyTx, female, 52 years)

The social role of the participants could be an environmental barrier as well. Participants indicated that when they feel as if they are required to fulfill a social role, for instance as a caretaker in their family, interference with being physically active can occur.

‘I would like to increase my physical activity level but then first the situation at home should be more stable, with my two sons and my wife. A lot of things happened and my primary goal is that I want me and my family to be okay again.’

(HeartTx, male, 51 years)

‘I have my physical limitations… my wife is taking care of our handicapped daughter for 19 years already… We always took care of her together but she does that on her own now. She needs a lot of care… So… when I would go cycling then there are so many other things [I could do]. I actually would leave my wife to it, so I rather stay at home and see what I can do.’

(KidneyTx, male, 54 years)

#### Physical environment

Bad weather is referred to as a barrier to PA by participants while having a dog is indicated as a motivation to being physically active. Both were classified as aspects from the physical environment. No reference was made to availability or accessibility of built and natural facilities by the participants.

#### Other

A specific aspect for the population under study is the use of immunosuppressant medication which is indicated by participants as a barrier to PA due to the side effects.

‘I wanted to do a contact sport but because of my new liver and the fact that I developed osteoporosis due to the use of medication, the risk was too high.’

(LiverTx, male, 22 years)

A lack of financial resources was mentioned as a barrier in a limited number of interviews (three out of sixteen).

## Discussion

This qualitative research identified the perceived barriers to and facilitators of PA in SOT recipients. The most common and important barriers were physical limitations, energy level, fear, and comorbidities. The most frequently indicated and important facilitators were motivation, coping, consequences of (in)activity, routine/habit, goals/goal priority, and the transplanted organ. Important neutral factors that acted as barriers or facilitators included self-efficacy and the expertise of personnel.

Several factors that were classified in the personal components: *physical* (physical limitations, lack of energy, co-morbidity, weight, age), *psychological* (motivation, coping, goals) and *other* (routine/habit, consequences of (in)activity) are also indicated as barriers and facilitators to PA in the general population and in older adults [35–38]. The same applies to the majority of factors classified in *social environment* (social support, group activity, social role), *physical environment* (weather) and *other* (financial resources) [35–38]. Although little studies are available, remarkable agreements in barriers to and facilitators of PA are seen with patients that underwent coronary artery bypass graft (CABG) surgery. In a recent qualitative study in CABG patients the most commonly cited barriers were ‘other time commitment’, ‘inclement weather’, and ‘pain/injury/illness related or unrelated to the surgery’ [39]. These most salient barriers, but also almost all other indicated barriers are present in the current study as well. The priority of the barriers, however, seems to be different for CABG patients and recipients of SOT. Physical limitations (pain/injury/illness related or unrelated to the surgery) are the number one barrier in recipients of SOT where it has a lower priority in CABG patients, as is also the case for energy level being a barrier. Where the weather is a highly salient barrier in CABG patients the priority was clearly lower in recipients of SOT. The most commonly mentioned facilitators in CABG patients were ‘it feels good to exercise’, and ‘improving physical health’ [39]. These most salient facilitators, but also the other indicated facilitators in the study are very similar to the indicated facilitators in the current study. The priority of facilitators also seems to concur. Furthermore, the items classified in the personal components *physical* and *psychological* and in the *social* and *physical environment* corresponding to those experienced in the general population also show many similarities with the barriers and facilitators in patients with end-stage organ disease [21–28]. It therefore seems likely that the majority of these barriers and facilitators are already present in the pre-transplant phase. Whether the number and/or severity of barriers and facilitators change from the pre-transplant phase to the post-transplant phase has not been reported so far.

Besides the barriers and facilitators similar to those experienced in the general population, CABG patients, and in the end-stage organ disease phase, there are several factors specific to SOT recipients. Perhaps the most striking is that several participants described to be inactive or moderately active before they became ill and during the period before transplantation, however, are now more active due to a feeling of responsibility to take good care of the received organ. Where earlier research demonstrated that previous exercise participation in adulthood is associated with current exercise participation [35,36,40], it seems that this association may not apply to all SOT recipients. Also associated with the transplanted organ but functioning as a barrier is a fear of damaging the new organ and insecurity with body signals. Therefore, it seems important that information and practical guidance are offered to recipients experiencing this barrier. The negative side-effects of immunosuppressants on muscle mass, muscle fiber type proportion and osteoporosis can lead to physical limitations and thereby function as a barrier to PA [17–19,41–43]. Related to the decreased muscle mass and the fiber type switch in combination with the usually prolonged period of reduced activity prior to and during hospitalization for transplantation, recipients often experience a lack of strength. This lack of strength could be a barrier and a facilitator, however, this needs specific attention in this population. A final distinct aspect mentioned is the expertise of personnel. It is indicated that (allied) health care providers should have sufficient knowledge about this particular population. Exercise specialists (e.g. expert physical therapists or clinical kinesiologists) could be deployed to reduce several barriers and utilize facilitators. With an adequate program and guidance, a reduction could likely be achieved in physical limitations, fear, side effects of medication, and weight as well as an increase in strength, energy level, reinforcement, and self-efficacy.

Results of the current study partly confirm the finding of the quantitative study on barriers to and facilitators of PA in kidney transplant recipients [29]. The major barriers in that study were ‘lack of motivation’, ‘preferring to spend time otherwise’, ‘weather’, ‘fatigue’, and ‘health conditions’. Major facilitators were health benefits and social support systems. The current study adds depth to these factors by providing more context and looking at why these barriers and facilitators are experienced. Furthermore, additional transplant specific barriers and facilitators are identified.

When taking all of the indicated barriers and facilitators into account, it seems that guidance for SOT recipients could best be initiated in an expert center or by specialized therapists. Depending on the amount and types of barriers, an interdisciplinary team or specialized exercise therapist could be deployed to initiate PA following transplantation. When sufficient knowledge and experience with training is gained, recipients are likely able to continue in their own environment. The most appropriate timing for this program might differ per person, type of transplantation, and rate of recovery after transplantation. As there were no clear differences between the transplant recipients’ groups, there appears to be no substantiation for treating these groups differently considering barriers to and facilitators of PA.

To our knowledge, this is the first study to investigate barriers to and facilitators of PA in a qualitative manner and between all SOT groups. Therefore, it provides new insight into the reasons and motives of PA behavior in this population and demonstrates that the various groups experience largely equal barriers and facilitators. A limitation of the study is that the current level of PA was not explicitly quantified [44,45] and that therefore no sub-analyses could be made on active versus non-active participants. The sample size was small as is common in qualitative research. Although, one could argue that sixteen participants, subdivided into four groups is rather limited. However, we checked for newly emerging themes to determine data saturation and the final four interviews did not yield any new themes. This appears to support the assumption that the concepts derived can be transferred to a wider population of SOT recipients. Nonetheless, some caution should be taken into account and further, quantitative, research should be undertaken to confirm these findings and to study the relevance of individual constructs per organ transplantation group on PA behavior.

## Conclusion

The identified barriers and facilitators are partly comparable to the general population and partly specific for SOT recipients. The overview of barriers and facilitators generated in this study indicates several personal and environmental factors that can be considered in intervention development. Further research should be undertaken to investigate if focusing on the proposed barriers and facilitators will result in increased physical activity behavior and reduced sedentary time in SOT recipients.

## Supporting Information

S1 Appendix Interview guide. (DOCX)

S1 Table Distribution of mentioned barriers and facilitators across transplant recipient groups. Tx, transplantation.
(DOCX)
