# Supplementary material for: Perceived Barriers to and Facilitators of Physical Activity in Recipients of Solid Organ Transplantation, a Qualitative Study
Source: PLoS One. 2016 Sep 13;11(9):e0162725. doi: 10.1371/journal.pone.0162725 (PMC5021267; doi:10.1371/journal.pone.0162725)
Supplement: S1 Appendix — (DOCX) [file pone.0162725.s001.docx]

**S1 Appendix. Interview guide**

- Warming up/informal conversation
- Explanation goal interview and introduction interviewer
- Informed consent (audio taping and use of data)
- Questionnaire personal details
- Explanation definition of physical activity

*Semi-structured interview*

- Can you describe how physically active you are during a regular day? (Job/commuting activities/ sports/ occupational activities)
- Are you satisfied with your current activity level? And if not, what would you like to change?
- Do you experience barriers to be physically active or to become more physically active?
- Were there any restrictions given by the medical doctor or the physical therapist in what you could do after your organ transplantation?
- Do you experience anxiety or restraint to perform certain activities, and if so, which activities and why?
- What motivates you to be physically active or to become more physically active?
- How did the organ transplantation influence your ability to be physically active?
- What could increase your participation in physical activity or sports?

*Closing question*

- Do you want to add something or did we miss something you did want to mention related to the topic of the interview?

*Examples of probe questions*

- Can you give an example? Could you explain that in more detail? How?
