# Supplementary material for: Perceived Barriers to and Facilitators of Physical Activity in Recipients of Solid Organ Transplantation, a Qualitative Study
Source: PLoS One. 2016 Sep 13;11(9):e0162725. doi: 10.1371/journal.pone.0162725 (PMC5021267; doi:10.1371/journal.pone.0162725)
Supplement: S1 Table — Tx, transplantation. (DOCX) [file pone.0162725.s002.docx]

**S1 Table. Distribution of mentioned barriers and facilitators across transplant recipient groups**

|  | Heart-tx  *n*=4 | Lung-tx  *n*=4 | Kidney-tx  *n*=4 | Liver-tx  *n*=4 | Total  *N*=16 |
| --- | --- | --- | --- | --- | --- |
| *Barriers* | | | | | |
| Physical limitations | 4 | 3 | 4 | 4 | 15 |
| Energy level | 2 | 2 | 4 | 4 | 12 |
| Fear | 3 | 2 | 2 | 2 | 9 |
| Comorbidity | 2 | 1 | 3 | 2 | 8 |
| Side-effects medication | 1 | 0 | 2 | 3 | 6 |
| Bad weather | 1 | 2 | 1 | 2 | 6 |
| Social role | 2 | 0 | 1 | 2 | 5 |
| Post-transplant life-events | 1 | 0 | 2 | 1 | 4 |
| Age | 2 | 0 | 0 | 1 | 3 |
| Financial resources | 1 | 0 | 2 | 0 | 3 |
| *Neutral* | | | | | |
| Self-efficacy | 3 | 3 | 3 | 4 | 13 |
| Expertise of personnel | 4 | 4 | 3 | 2 | 13 |
| Group activity | 2 | 0 | 1 | 1 | 4 |
| *Facilitators* | | | | | |
| Motivation | 4 | 4 | 4 | 4 | 16 |
| Goals/goal priority | 4 | 4 | 3 | 4 | 15 |
| Coping | 4 | 3 | 4 | 4 | 15 |
| Routine/habit | 4 | 4 | 3 | 3 | 14 |
| Consequences of (in)activity | 3 | 3 | 4 | 4 | 14 |
| Transplanted organ | 3 | 3 | 3 | 1 | 10 |
| Social support | 1 | 3 | 2 | 3 | 9 |
| Strength | 2 | 2 | 2 | 2 | 8 |
| Weight | 1 | 0 | 2 | 2 | 5 |

Tx, transplantation
